# JBI Critical appraisal checklist for systematic reviews and research syntheses

**DOI:** 10.29173/jchla29801

**Published:** 2024-12-01

**Authors:** Mackenzie Hilton

**Affiliations:** Librarian CAMH Toronto, ON, Canada

**Product:** JBI *Critical appraisal checklist for systematic reviews and research syntheses*

**URL:** https://jbi.global/sites/default/files/2019-05/JBI_Critical_Appraisal-Checklist_for_Systematic_Reviews2017_0.pdf

## Purpose

The purpose of this product review is to highlight the Joanna Briggs Institute’s (JBI) *Critical appraisal checklist for systematic reviews and research syntheses*. This review will elucidate the strengths and weaknesses of the checklist, and indicate scenarios where use of the checklist may be warranted.

## Product description

JBI’s *Critical appraisal checklist for systematic reviews and research syntheses* provides a quick and efficient way to evaluate research methodologies and identify attempts made by authors to reduce bias. By asking 11 questions about a review’s methodology, search strategy, and bias, JBI’s critical appraisal checklist informs the “synthesis and interpretation” of studies [1]. The checklist serves as a tool to improve the transparency, reliability, and accuracy of umbrella reviews by appraising the quality and reliability of included studies.

## Intended users

The checklist is intended for researchers conducting umbrella reviews or other types of evidence synthesis that appraise systematic reviews and research syntheses.

## Questions asked by the checklist

Checklist sub-questions are the author’s summary of the checklist description:

1. *Is the review question clearly and explicitly stated?*
  - How/where is the research question stated?
  - Is the research question stated in a protocol?
  - Is the research question formulated around PICO?
2. *Were the inclusion criteria appropriate for the review question?*
  - Are inclusion criteria provided?
  - Do the inclusion criteria match the research question?
  - Do the inclusion criteria reflect the PICO components?
  - Are relevant study types included?
3. *Was the search strategy appropriate?*
  - Is a search strategy provided?
  - Does the search strategy address each PICO component?
  - Were relevant and logical keywords searched for?
  - Were subject headings searched for in conjunction with keywords?
  - Were limits and search filters justified?
4. *Were the sources and resources used to search for studies adequate?*
  - Were the sources searched comprehensively?
  - Were multiple electronic databases searched?
    - Were major bibliographic databases searched? (Medline and CINAHL are specifically mentioned)
    - Were relevant subject-specific databases searched? (i.e., the PEDro database for questions about physical therapy interventions, or ERIC for educational interventions)
  - Was grey literature or “unpublished studies” searched for?
5. *Were the criteria for appraising studies appropriate?*
  - Were the studies critically appraised?
  - What were the methods/tools/ instruments used for appraisal?
  - Were the methods/tools/instruments used for appraisal appropriate?
6. *Was critical appraisal conducted by two or more reviewers independently?*
7. *Were there methods to minimize errors in data extraction?*
  - Were extractors trained?
  - What methods/tools/instruments were used for extractions?
8. *Were the methods used to combine studies appropriate?*
9. *Was the likelihood of publication bias assessed?*
  - Were statistical tests used to assess the potential for publication bias?
10. Were recommendations for policy and/or practice supported by the reported data?*
  - Are there recommendations for policy and practice?
  - Are the recommendations supported by the results?
  - Have the strength of the findings and the quality of the research been considered in the recommendations?
11. Were the specific directives for new research appropriate?*
  - Is there an indication of future research directions?

*Questions 10-11 assess the overall quality of the review, whereas questions 1-9 assess the potential for bias within the research methodology.

## Compatibility

JBI’s *Critical appraisal checklist for systematic reviews and research syntheses* can be accessed in any internet browser, and on any device that has internet access. The checklist can be freely downloaded in PDF format. The PDF can then be viewed and filled using Adobe Acrobat or other PDF viewing programs.

The checklist is also compatible with review software, such as DistillerSR. The checklist can be incorporated into a project page that allows teams to collaborate toward the appraisal of studies.

## Usability

The guiding document (attached to the checklist and linked above) “JBI critical appraisal checklist for systematic reviews and research syntheses,” by Aromataris et al., explains how to answer each question on the checklist. The importance of each question is described, as well as what to consider when answering. Although the checklist is easy to use, it requires critical thinking and subjective evaluation from the user.

## Strengths and weaknesses

### Strengths

- Free, easy, and quick to access, use, and share
- Evaluation criteria are clearly stated and described
- Can easily be incorporated into review software, such as DistillerSR
- Questions are relevant and specific
- Questions are thorough and sufficiently address the target areas of bias and review quality
- The guiding document attached to the checklist provides additional instructions and guidance

### Weaknesses

- The questions asked could be more comprehensive
  - E.g. A question about conflicts of interest
  - E.g. A prompt to consider whether advanced search strategies (adjacency, truncation, etc.) were used
- Sacrifices some speed due to the critical thinking needed to answer some questions

## Currency

JBI’s critical appraisal checklist was created in 2017. Although the checklist is a few years old, the checklist asks relevant questions for reviews conducted recently. JBI also provides a *Manual for evidence synthesis* that was updated in 2024 [2]. This *Manual for evidence synthesis* utilizes the same critical appraisal checklist, attesting to the checklist’s relevancy.

## Comparison with similar products

The Critical Appraisal Skills Program (CASP) provides a *Systematic review checklist* with comparable features to the one offered by JBI. These similarities and differences are captured in Table 1.

**Table 1:** Comparison of JBI’s critical appraisal checklist and the Critical Appraisal Skills Program (CASP)

Feature	JBI’s critical appraisal checklist	CASP systematic review checklist
**# of questions**	11 questions	10 questions
**Additional support**	Detailed instructions and points to consider for each question Associated publications for additional support *JBI manual for evidence synthesis*(2024)	Includes “hints,” or points to consider for each question “How to use the CASP checklist” contains basic information about using a CASP checklist, but is not specific to the *Systematic review checklist* Offers workshops and email support for critical appraisal
**Appropriateness of questions**	Questions primarily aim to uncover any potential for bias Questions 1-9 focus on steps of the research methodology where bias could be introduced Questions 10-11 assess the quality of a review Questions are appropriate for appraising the bias and overall quality of a review Some questions are subjective, which could introduce bias in the appraisal process What is considered “appropriate” may vary	Questions focus more on the results of a review, and less on bias Questions are appropriate for appraising the results of a review only Some questions are vague and introduce a greater potential for bias in the appraisal process
**Are comment boxes provided?**	Yes, 1 box for comments is located **at the end of the form**	Yes, 1 box for comments is located **after each question**
**Downloadable**	Downloadable as a PDF	Downloadable as a PDF and Word document
**Ease of use**	Very easy and intuitive to use	Very easy and intuitive to use
**Institutional affiliation**	Joanna Briggs Institute (JBI) “A recognized global leader in evidence-based healthcare [1]	Critical Appraisal Skills Programme (CASP) “Over 25 years of delivering training to healthcare professionals.”[3]
**Response options**	Yes No Unclear Not applicable	Yes Can’t tell No
**Requirements and cost**	Free to access	Free to access
**User interface**	Checkboxes and textboxes	Checkboxes and textboxes
**What is assessed?**	Potential for bias in a review Indicators of review quality	Are the results of a study valid? What are the results? Can the results be applied to the local population?

## Conclusion

JBI’s *Critical appraisal checklist for systematic reviews and research syntheses* offers researchers a quick and efficient way to evaluate the research methodologies of systematic reviews. The checklist serves as a guide by asking the user 11 questions. These questions pertain to the research question, search strategy, resources searched, inclusion and exclusion criteria, data extraction, appraisal, publication bias, accuracy of the results, and future directions. The questions asked are thorough and sufficiently appraise the target areas of bias and the overall quality of a systematic review. JBI’s *Critical appraisal checklist for systematic reviews and research syntheses* has an advantage over similar checklists in light of its precise scope, thoroughness of questions, and supporting documents.
